# A repeat-dose thorough QT study of inhaled fluticasone furoate/vilanterol combination in healthy subjects

**DOI:** 10.1111/bcp.12243

**Published:** 2014-02-21

**Authors:** Rodger Kempsford, Ann Allen, Kathryn Kelly, Parminder Saggu, Courtney Crim

**Affiliations:** 1 Clinical Pharmacology, Global Clinical Respiratory Medicines Development Centre, GlaxoSmithKlineStevenage, UK; 2 Department of Clinical Pharmacology, Modelling and Simulation, GlaxoSmithKlineStevenage, UK; 3 Department of Clinical Pharmacology Science and Study Operations, GlaxoSmithKlineStockley Park, UK; 4 Department of Clinical Statistics, BetaplexLondon, UK; 5 Global Clinical Respiratory Medicines Development Centre, GlaxoSmithKlineResearch Triangle Park, USA

**Keywords:** fluticasone furoate, healthy subjects, moxifloxacin, thorough QT, vilanterol

## Abstract

**AIMS:**

This study was designed as a thorough QT (TQT) study to evaluate the effects of fluticasone furoate(FF)/vilanterol (VI) in healthy subjects. Supportive data from a TQT study conducted with FF are also presented.

**METHODS:**

This was a randomized, placebo-and positive-controlled, double-dummy, double-blind, four-way crossover study, in which healthy subjects (*n* = 85) were randomized to 7 days of once-daily treatment of FF/VI (200/25 or 800/100 μg) or placebo or single-dose oral moxifloxacin (single-blind, 400 mg). In the supportive TQT study, subjects (*n* = 40) were randomized to single-dose inhaled FF(4000 μg), oral moxifloxacin (400 mg) or placebo.

**RESULTS:**

There was a lack of effect of FF/VI (200/25 μg) on QTcF (Fridericia's correction); all time-matched mean differences from baseline relative to placebo (0–24 h) were <5 ms, with upper 90% confidence intervals (CI) of <10 ms. At 800/100 μg, FF/VI had no significant clinicaleffect on QTcF except at 30 min postdose when the 90% CI was >10 ms [mean (90% CI), 9.6 ms (7.2, 12.0)]. No effect on QTci (individually corrected) was observed at either strength of FF/VI, with mean time-matched treatment differences <5 ms at all time points [upper 90% CIs <10 ms (0–24 h)]. Assay sensitivity was confirmed; moxifloxacin prolonged QTcF and QTci, with time-matched mean differences from baseline relative toplacebo of >10 ms (1–8 h postdose).

**CONCLUSIONS:**

Repeat once-daily dosing of FF/VI (200/25 μg), which is the highest therapeutic strength used in phase III studies, is not associated with QTc prolongation in healthy subjects. Supratherapeutic strength FF/VI (800/100 μg) demonstrated a small transient effect on QTcF but not on QTci.

**WHAT IS ALREADY KNOWN ABOUT THIS SUBJECT**

- Fluticasone furoate (FF)/vilanterol (VI) inhalation powder is being developed as a fixed-dose combination inhaled corticosteroid/long-acting β_2_-agonist for chronic obstructive pulmonary disease and asthma.
- Thorough QT (TQT) studies are required to evaluate the potential for QT/corrected QT (QTc) prolongation of all new non-antiarrhythmic drugs that are systemically bioavailable.
- Supratherapeutic doses of inhaled long-acting β_2_-agonists have the potential to produce systemic pharmacodynamic effects, including prolongation of the QT interval.

**WHAT THIS STUDY ADDS**

- This study describes the effect on the QTc interval [Fridericia's correction (QTcF) and individually corrected (QTci)] at therapeutic and supratherapeutic strengths of FF/VI.
- The results show that at a FF/VI therapeutic strength (200/25 μg) there was no prolongationof time-matched QTcF or QTci intervals.
- At an FF/VI strength of 800/100 μg, representing four times greater than the highest therapeutic strength used in phase III studies, there was a small transient prolongation of time-matched QTcF, while QTci wasnot prolonged.

## Introduction

International guidelines advocate the use of inhaled long-acting β_2_-agonists (LABA) in combination with inhaled corticosteroids (ICS) for the maintenance therapy of asthma in patients who remain symptomatic despite use of low-medium dose ICS alone [1,2]. ICS/LABA combinations are also recommended for the treatment of chronic obstructive pulmonary disease (COPD) and have been shown to be more effective than the individual components [3].

Fluticasone furoate (FF) inhalation powder is a once-daily ICS shown to be effective for treating mild-to-moderate asthma in patients [4–7] including those who remain symptomatic on nonsteroidal therapy [4] or on medium-dose ICS [6]. Vilanterol inhalation powder (VI) is a once-daily inhaled LABA that has demonstrated prolonged bronchodilatation of at least 24 h and is well tolerated in patients with asthma who remain symptomatic despite controller medication [8,9] and in COPD patients [10] at doses up to and including 50 μg. Fluticasone furoate/vilanterol inhalation powder is in development as a once-daily combination for maintenance treatment of asthma (FF/VI 200/25 or 100/25 μg) and COPD (100/25 μg). Currently available ICS/LABA combinations, such as fluticasone propionate/salmeterol and budesonide/formoterol, require twice-daily administration [11,12]. Once-daily treatments may help to improve patient compliance and, therefore, overall disease management of these chronic conditions [12–15].

Inhaled β_2_-agonists have been associated with class-related systemic pharmacodynamic effects, such as the potential to raise heart rate and cause ventricular arrhythmias in patients with obstructive airways disease [16,17]. Thorough QT (TQT) studies are recommended to quantify the effects of systemically bioavailable, non-antiarrhythmic drugs on the QT/corrected QT (QTc) interval; it is recommended that these are conducted in healthy subjects rather than patients at higher risk of arrhythmias [18,19]. In this paper, we describe the TQT study conducted with FF/VI administered via a dry powder inhaler (DPI). This study was conducted to determine whether FF/VI 200/25 μg (representing the highest strength assessed in phase III studies in asthma and COPD) and a fourfold multiple of this, FF/VI 800/100 μg, both administered once daily for 7 days, were associated with a clinically significant effect on QTc. Supportive data from the TQT study conducted with single-dose inhaled FF administered via the Diskus™/Accuhaler™ inhaler (GlaxoSmithKline) are also presented. This FF TQT study was performed to support the development of both the intranasal formulation and inhaled formulation FF products and is included here to help to ascertain whether any effects seen with FF/VI were to be attributed to either the FF or VI components.

## Methods

### Fluticasone furoate/vilanterol TQT study

#### Subjects

Eighty-five healthy nonsmokers (i.e. not smoked within 6 months; pack history ≤ 10 years) aged 18–65 years, with a body mass index within the range 18.5–29 kg m^−2^, forced expiratory volume in one second (FEV_1_) ≥ 85% of predicted values 20 and no history of or no current significant abnormalities in relationship to ECG (PR interval > 210 ms; ventricular rate, <40 beats min^−1^; mean QTcF (Fridericia's correction) ≥ 450 ms; Q waves ≥ 30 ms; QRS < 60 ms and ≥ 120 ms), vital signs or clinical laboratory assessments at baseline were included. Subjects were excluded if they had used topical corticosteroids within 8 weeks or inhaled or intranasal corticosteroids within 4 weeks of screening. Written informed consent was obtained from each subject prior to the conduct of any study-related procedure.

#### Study design

This was a randomized, placebo-controlled, double-dummy, four-way crossover study conducted at a single study site (Hammersmith Medicines Research, UK) between 23 June 2010 and 4 January 2011. The study was conducted in accordance with the ethical principles of the Declaration of Helsinki and approved by the independent ethics committee of the participating centre prior to the start of the study. The study comprised a screening period (up to 28 days), four repeat-dose treatment periods (7 days each to ensure that FF and VI were at steady state) separated by at least 7 days washout, and a follow-up visit conducted within 14 days of the last dose. Subjects were randomized (1:1:1:1) to one of four treatment sequences, receiving the following treatments in random order: FF/VI (200/25 μg, therapeutic strength as one inhalation of 200/25 μg and one placebo inhalation); FF/VI (800/100 μg, supratherapeutic strength as two inhalations of 400/50 μg); matching placebo (two placebo inhalations); or moxifloxacin tablet (400 mg, therapeutic dose). Note that FF/VI was administered as 200/25 and 800/100 μg and represented emitted doses from the DPI of 184/22 and 736/88 μg, respectively. Moxifloxacin (Avelox®; Bayer) [21] was included as the positive control to determine assay sensitivity [22]. For the inhaled treatment arms, FF/VI or matching placebo [both from GlaxoSmithKline (GSK)] were administered once daily in the morning for 7 days under double-blind conditions via DPI. The DPI was a dual-strip dry powder inhaler that contained FF in one strip and VI in the second strip for the FF/VI products, or placebo in both strips. On day 7, a nonmatching placebo tablet (for moxifloxacin; GlaxoSmithKline) was administered in single-blind conditions. During the moxifloxacin treatment period, subjects received double-blind inhaled placebo on days 1–7 (inclusive) and a single-blind moxifloxacin tablet on day 7. Dosing was supervised on treatment days 1 and 7 and was unsupervised on days 2–6 (see online Supporting information). The central randomization schedule was generated by GSK Discovery Biometrics using validated internal software. The investigator or designee received the medication assignment information and randomized the subjects using sequential randomization numbers assigned in chronological order; the randomization and treatment period numbers were printed on the labels for the product. Subjects abstained from taking prescription or nonprescription drugs (except paracetamol, ≤2 g day^−1^) from 7 days (or five half-lives) prior to screening through to follow-up.

#### Assessments of ECG and evaluation of QTc

At screening, 24 h Holter ECG recordings were taken to exclude underlying cardiac arrhythmias. At all other time points, 12-lead continuous ECG digital monitoring (Mortara H12plus, 1000 Hz Holter monitor) was performed before any other study assessments scheduled at the same nominal time point. To account for intrinsic variability, triplicate measurements were taken at all time points, with the mean QTc value taken from five separate beats analysed on each ECG; QTc for an individual beat was calculated from the preceding RR interval. ECG measurements were taken over 12 h on day −1 (treatment period 1 only), predose only (day 1) and on day 7 predose, 5, 10 and 30 min, 1, 2, 4, 8, 12, 16 and 24 h postdose. Subjects were in a semi-supine position (having rested for 30 min) for the first reading up to and including the 4 h time point, then supine or semi-supine for the remaining time points. On ECG assessment days, dosing was conducted in the fasted state and subjects continued fasting after dosing for 1 h (day 1) and 4 h (day 7). Critical ECG data were not collected immediately after meals or during sleep; meals were administered immediately after scheduled ECG collection time points; water (at room temperature) was allowed except for 1 h either side of dosing.

The ECG readings were transmitted electronically to an independent, blinded central cardiologist for digital calliper measurement of conduction intervals (Quintiles, India). A lack of clinically significant effect was defined in accordance with the International Conference on Harmonisation (ICH) E14 guidelines as the upper 90% confidence interval (CI) limits for mean time-matched QTc differences from placebo being <10 ms [18,19].

The QT intervals were corrected using Fridericia's (QTcF), Bazett's (QTcB) and individual correction factors (QTci) [23–27]. The QTci was calculated using all baseline data from day −1, day 1 (predose) and all placebo (i.e. nontreatment) data. A linear regression model (QT = α + β × RR) was fitted for each subject, and the slopes (β) for the regression models were estimated. Individual QT values were subsequently corrected using the formula: QTci = QT + β(1 − RR), where β is the estimate of the correction factor obtained from the first step from the respective model [27]. As QTcB does not correct very well when heart rates are changing, QTcB was not analysed further.

#### Pharmacokinetic assessments

Blood samples (6 ml) were taken on day 7 of each treatment period predose, then at 5, 10 and 30 min, 1, 2, 4, 8, 12, 16 and 24 h postdose to quantify steady-state concentrations of FF and VI during ECG assessment and investigate any relationship between plasma concentration and QTc values for VI and FF. Samples taken after administration of moxifloxacin were to be analysed only if an unexpected or unusual QTc response was recorded. Analysis of plasma samples was performed using validated methods based on solid-phase extraction followed by high-performance liquid chromatography–tandem mass spectrometry. Lower limits of quantification were 10 pg ml^−1^ for FF and VI; the higher limit of quantification was 1000 pg ml^−1^ for FF and 10 000 pg ml^−1^ for VI. Quality controls prepared at three different concentrations analysed with each batch of samples met the acceptance criteria of no more than one-third of the quality control results deviating from the nominal concentration by >15%, with at least one quality control result acceptable at each concentration. Standard noncompartmental methods using WinNonlin (version 5.2) were used to derive pharmacokinetic (PK) parameters including maximum observed concentration (*C*_max_), time to maximum concentration (*T*_max_) and area under the concentration-time curve from time zero to 24 h post-dose (AUC_(0–24)_).

#### Safety assessments

Adverse events (AEs), vital signs (blood pressure and heart rate), 12-lead ECG, spirometry (FEV_1_) and clinical laboratory assessments (clinical chemistry, haematology and urinalysis) were monitored at intervals during the study.

#### Statistical analysis

##### Sample size

The sample size calculation was performed using simulated data based upon the comparison of FF/VI 200/25 μg with placebo in terms of the change from baseline in the QTcF interval using an integrated analysis of previous studies with VI (see online Supporting information and Supporting information,Table S1); FF is not associated with QT effects; therefore, sample size calculations were based on VI only.

In order to rule out an effect size of ≥10 ms with overall 90% power at the one-sided 5% significance level, assuming a true treatment difference of 3 ms, between-subject SD of 15 ms, within-subject SD of 10 ms, and a within-subject correlation between values from any two time points of 0.55, 66 evaluable subjects were required.

##### Statistical methods

The ‘per protocol’ population was the primary population of interest for the analysis of the primary end-point data, defined as subjects having received at least six doses of study medication out of seven, including day 7, for each of the four treatment periods; the PK population was defined as subjects in the ‘all subjects’ population (defined as all subjects who received at least one dose of study medication) for whom a PK sample was obtained and analysed; this was used for all PK analyses.

The primary end-point was change from baseline in QTcF interval at each of the time points on study day 7 for FF/VI 200/25 μg compared with time-matched placebo. The primary end-point and change from baseline in secondary end-point ECG parameters (QTci and heart rate), at each time point on day 7, were analysed using a repeated-measures analysis of covariance (ANCOVA) model, fitting subject as a random effect, and period, time, treatment and time-by-treatment interaction as fixed-effect terms. Subject and period baseline were also included as continuous covariates, and the interaction term for period baseline by time was also fitted. Time was fitted as a repeated effect using an unstructured variance–covariance R-matrix.

Categorical analysis of QTcF was performed to determine the number and percentage of subjects per treatment who had an increase from baseline on day 7 of <30 ms, 30 to <60 ms, and ≥60 ms. In addition, the number and percentage of subjects per treatment who had a postdose QTcF on day 7 of ≤450 ms, >450 to ≤480 ms, >480 to ≤500 ms, and >500 ms were summarized.

#### Pharmacokinetic–pharmacodynamic (PK-PD) modelling

The QTcF and heart rate values showed significant effects for the supratherapeutic FF/VI strength; hence, PK-PD analyses focused on these end-points.

The relationship between PK and PD parameters was initially investigated by scatter plots of individual time-matched differences from placebo in changes from baseline for QTc and heart rate against FF and VI concentrations. Placebo data were expressed as *C*_max_ values of 0 pg ml^−1^. Pharmacokinetic–pharmacodynamic plots of mean time-matched differences from placebo in changes from baseline for QTc and heart rate against median FF and VI concentrations were also produced.

Evaluation of PK-PD for FF systemic exposure in the FF/VI study alone was confounded by the dosing of FF/VI inhalation powder as a fixed-ratio combination. As FF systemic exposure increased with dose, so did VI exposure. In the FF study, FF administered alone at high dose was shown to have no effect on QTcF or heart rate. Inclusion of data from this study in the scatter plots showed there to be no relationship between FF concentration and either QTcF or heart rate effect. Moreover, inhaled corticosteroids as a class are not associated with cardiovascular effects, whereas LABAs are [28]. Therefore, no formal PK-PD analysis was conducted for FF systemic exposure, and any effects seen in this study were considered to be related to VI; hence, PK-PD modelling was conducted using VI *C*_max_ data.

Population modelling of PK-PD was performed using mixed-effect modelling with the computer program NONMEM version 7 running in the Predictive Modelling Environment (PME), a UNIX server-based environment for NONMEM analysis. The method selected for minimization was First Order Conditional Estimation method with Interaction (FOCEI) [29]. Supporting application interfaces for data handling, exploratory diagnostics and simulation included Xpose V4 and various graphical software [30].

Model evaluation to assess the adequacy of the final model, including the effects of statistically significant covariates, was performed using a visual predictive check procedure as follows: 500 replicates of the original data set were simulated, based on the parameter estimates of the final model, and a 95% prediction interval computed based on the simulated data sets [31]. The observed concentration *vs*. time data were plotted on the prediction interval to assess visually the concordance between the simulated and observed data.

### Fluticasone furoate TQT study

#### Subjects

A similar study population was enrolled in the FF TQT study as for the FF/VI TQT study. Forty healthy nonsmokers (i.e. not smoked within 12 months; pack history ≤ 5 years) with a body mass index within the range of 19–31 kg m^−2^ (weight ≥ 50 kg) and ECG requirements of PR interval < 240 ms; ventricular rate > 45 beats min^−1^; mean QTcF < 430 ms (male) and < 450 ms (female); and Q waves < 40 ms or depth < 0.4–0.5 mV, were enrolled.

#### Study design

This study was similar to the FF/VI study but was a single-dose crossover study conducted at a single site (PAREXEL, UK) from 7 March to 7 June 2005 and approved by Brent Medical Ethics Committee. The following four treatments were given: inhaled FF (4000 μg); inhaled placebo (lactose); inhaled placebo [lactose with the excipient cellbiose octaacetate (COA; 6.25 mg)]; and oral moxifloxacin (400 mg), all supplied by GSK. The inhaled treatments were administered via Diskus™/Accuhaler™ inhaler. Results for the lactose placebo with COA were not significantly different from the lactose-alone treatment and are not presented in this paper because COA is not an excipient in either the FF or the FF/VI formulation. Subjects remained resident at the study site from the evening of day −1 until 24 h after dosing on day 1 (see online Supporting information). The central randomization schedule was generated by the GSK statistical department using validated internal software.

#### Assessments of ECG and evaluation of QTc

Triplicate ECG readings (each reading taken 2 min apart) were taken predose and at 15, 30 and 45 min, 1, 1.5. 2, 3, 4, 6, 12 and 24 h postdose, with subjects having rested in a supine position for 5 min prior to the first reading. Data for QTcF and QTcB were collected, but given the similarity between the data, only QTcF results are reported in this manuscript to provide a comparison with the FF/VI study.

#### Pharmacokinetic assessments

A sparse PK sampling approach was used (seven samples per subject per treatment period). Blood samples (4 ml) were taken predose and then one sample was taken during each of the following postdose time windows: 10–20 min, 1–2, 3–4, 6–8, 11–12 and 24 h. Population PK analysis was conducted using nonlinear effect modelling to derive PK parameters [30].

#### Safety assessments

In addition to the safety assessments described for the FF/VI study, lung function assessments (forced vital capacity and peak expiratory flow) were assessed.

#### Statistical analysis

##### Sample size

For the comparison of FF *vs*. placebo, a sample size of 32 evaluable subjects was calculated based on the requirement to show non-inferiority with regard to the primary end-point, the maximal mean change from baseline QTcF. This ruled out an effect size of >7.5 ms. Different criteria for calculating sample size were used in this study compared with the FF/VI study due to an update in the ICH E14 guidelines immediately prior to the conduct of the FF study and ensured that the FF study also analysed the mean change from baseline in QTcF at each time point (as it was analysed in the FF/VI study) as well as the maximal QTcF [18,19].

##### Statistical methods

Change from baseline in QTcF was analysed using an ANCOVA model with treatment, period, sequence, time and treatment-by-time interaction fitted as fixed effects and subject fitted as a random effect. Baseline QTcF was fitted as a continuous covariate. Time was fitted as a repeated effect using a heterogeneous autoregressive covariance structure. Categorical QTcF analysis was performed, as for the FF/VI study. The ‘all subjects’ population, defined as all subjects randomized to treatment who received at least one dose of study treatment (including placebo), was used for all safety and ECG analyses.

#### Pharmacokinetic–pharmacodynamic modelling

As no effect of FF on ECG parameters was observed, the relationship between ECG parameters and systemic exposure was not explored.

## Results

### Fluticasone furoate/vilanterol TQT study data

#### Demographics

Table 1 summarizes the subject disposition and demographics for the FF/VI study. Eighty-five subjects were randomized to treatment, of whom 73 subjects met the criteria for the per protocol population and 82 met the criteria for the PK population. Two subjects were withdrawn due to protocol violations relating to positive drugs of abuse tests, one subject prior to treatment period 2 (FF/VI 200/25 μg) and one subject during treatment period 1 (placebo).

**Table 1:** Subject demographics and baseline characteristics of study population for fluticasone furoate (FF)/vilanterol (VI) and FF studies (‘all subjects’ population)

	FF/VI TQT	FF TQT
**Number of subjects randomized (*n*)**	85	40
**Number of subjects completed [*n* (%)]**	77 (91)	39 (98)
**Number of subjects withdrawn [*n* (%)]**	8 (9)	1 (2.5)
**Reason for withdrawal**		
** Adverse events/serious adverse events**	0	0
** Withdrew consent**	5 (6)	NA
** Protocol violation**	2 (2)	NA
** Investigator discretion**	1 (1)	NA
** Other**	NA	1 (2.5)
**Age (years; median [range])**	28.0 [18–65]	29.1 [19–58]
**Gender [*n* (%)]**		
** Male**	49 (58)	22 (55)
** Female**	36 (42)	18 (45)
**Body mass index [kg m^−2^; mean (SD)]**	23.86 (2.87)	24.00 (2.73)
**Height [cm; mean (SD)]**	169.7 (8.5)	171.9 (6.7)
**Weight [kg; mean (SD)]**	68.95 (11.22)	71.19 (11.37)
**Race [*n* (%)]**		
** White Caucasian**	60 (71)	32 (80)
** African American/African heritage**	9 (11)	3 (8)
** Asian – Japanese/East/South East Asian heritage**	4 (5)	NA
** Asian – Central/South Asian heritage**	10 (12)	1 (2.5)
** Other**	2 (2)	4 (10)

Abbreviations are as follows: NA, not applicable; and TQT, thorough QT.

#### Pharmacodynamics

##### Effect of QT correction factor

Scatter plots of the uncorrected and corrected QT intervals against RR intervals were produced to check the effectiveness of each of the correction factors in correcting for heart rate (Supporting information, Figure S1A–D). The uncorrected QT interval correlated positively with the RR interval, indicating the need to apply a correction factor (Supporting information, Figure S1A). Bazett's correction did not adequately correct the QT interval and in fact, overcorrected it, resulting in a negatively correlated QTcB *vs*. RR interval (Supporting information, Figure S1B). In contrast, both QTcF and QTci adequately corrected the QT interval for changes in the RR interval, with no evidence of overcorrection or undercorrection (Supporting information, Figure S1C and D, respectively).

##### Effect of FF/VI and moxifloxacin on QTcF

**Figure 1:**
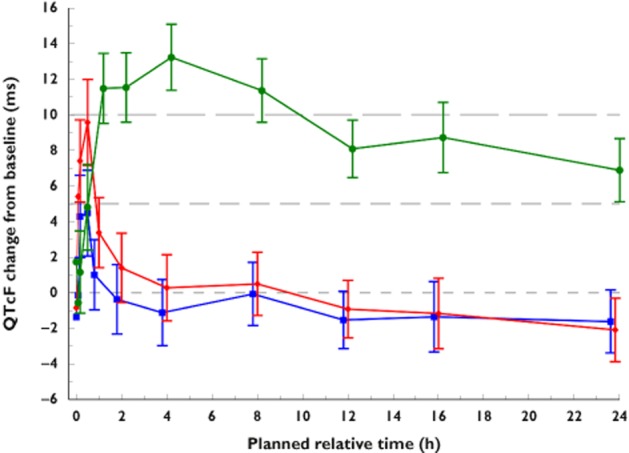
QTcF (Fridericia's correction) adjusted mean (90% CI) change from baseline (difference from placebo) 0–24 h after dosing on day 7 with FF/VI (800/100 and 200/25 μg) and single dose moxifloxacin (400 mg) (per protocol population). 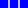, FF/VI 200/25 μg; 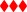, FF/VI 800/100 μg; 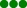, moxifloxacin 400 mg

Repeat dosing of the therapeutic strength of FF/VI (200/25 μg) showed a lack of effect in time-matched QTcF compared with placebo (Figure 1). The upper limit of the 90% CIs for the treatment differences did not exceed 10 ms at any time point over 24 h with all mean differences <5 ms. After repeat dosing with the supratherapeutic strength of FF/VI (800/100 μg), a slight and transient prolongation of QTcF was observed relative to placebo within the first hour of dosing; the largest mean [90% CI] increase occurred at 30 min postdose (9.6 ms [7.2, 12.0]). This was the only time point at which the 90% CI exceeded 10 ms.

Single-dose moxifloxacin (400 mg) produced an increase in time-matched QTcF compared with placebo, with mean treatment differences >10 ms seen at 1, 2, 4 and 8 h postdose (Figure 1). Mean treatment differences exceeded 5 ms at all time points, with all 90% CIs exceeding 5 ms from 1 to 24 h postdose. The highest mean change from baseline (13.2 ms) was seen at 4 h postdose.

##### Effect of FF/VI and moxifloxacin on QTci

**Figure 2:**
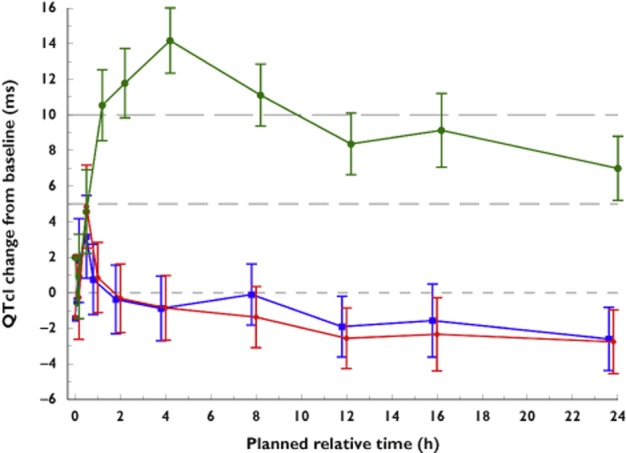
QTci (individually corrected) adjusted mean (90% CI) change from baseline (difference from placebo) 0–24 h after dosing on day 7 with FF/VI (800/100 and 200/25 μg) and single-dose moxifloxacin (400 mg) (‘all subjects’ population). 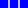, FF/VI 200/25 μg; 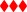, FF/VI 800/100 μg; 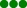, moxifloxacin 400 mg

Repeat dosing of the therapeutic strength of FF/VI (200/25 μg) showed a lack of effect in time-matched QTci compared with placebo (Figure 2). The upper limit of the 90% CIs for the treatment differences did not exceed 10 ms at any time point over 24 h, with all mean differences <5 ms. After repeat dosing with the supratherapeutic strength of FF/VI (800/100 μg) there was also a lack of effect on QTci; the upper limit of the 90% CIs for the treatment differences did not exceed 10 ms at any time point over 24 h, with all mean differences <5 ms (Figure 2). The mean QTci values (difference from placebo) were comparable at all time points for the two treatments (Figure 2).

Single-dose moxifloxacin (400 mg) produced an increase in time-matched QTci compared with placebo that was comparable with the QTcF results; mean treatment differences were >10 ms at 1, 2, 4 and 8 h after dosing and >5 ms at all time points (1–24 h after dosing); all lower bound 90% CIs exceeded 5 ms over the same time period (Figure 2).

##### Categorical QTcF analysis

There were no absolute QTcF values >450 ms after repeat dosing with FF/VI 200/25 or 800/100 μg, while changes in QTcF of >30 ms were seen in only three subjects (4%) after FF/VI 800/100 μg (Table 2).

**Table 2:** Summary of maximal QTcF (Fridericia's correction) postdose and maximal change from baseline in QTcF (per protocol population)

Treatment	QTcF maximum postdose (ms)				QTcF maximal change from baseline (ms)			
	≤450	>450–480	>480–500	>500	≤30	≥30–60	≥60
	[*n* (%)]	[*n* (%)]	[*n* (%)]	[*n* (%)]	[*n* (%)]	[*n* (%)]	[*n* (%)]
**FF/VI 200/25 μg**	73 (100)	0	0	0	73 (100)	0	0
**FF/VI 800/100 μg**	73 (100)	0	0	0	70 (96)	3 (4)	0
**Placebo**	73 (100)	0	0	0	73 (100)	0	0
**Moxifloxacin 400 mg**	69 (95)	4 (5)	0	0	61 (84)	12 (16)	0

Single-dose moxifloxacin produced absolute QTcF values >450–480 ms in a minority of subjects (5%), with no values >480 ms. Changes in QTcF over 30 ms relative to baseline were observed in 12 (16%) subjects (Table 2), with no values ≥60ms.

##### Heart rate

Repeated doses of therapeutic and supratherapeutic strengths of FF/VI were associated with changes in heart rate; maximal effects were seen 10 min postdose for both strengths (Table 3). Single-dose moxifloxacin (400 mg) was associated with heart rate changes of <3 beats min^−1^ in time-matched heart rate (mean change from baseline) compared with placebo.

**Table 3:** Mean change from baseline for heart rate on day 7 (all subjects population)

Time point	Least square means (beats min^−1^)				Treatment difference [90% CI] (beats min^−1^)		
	FF/VI 200/25 μg	FF/VI 800/100 μg	Placebo	Moxifloxacin 400 mg	FF/VI 200/25 μg – placebo	FF/VI 800/100 μg – placebo	Moxifloxacin 400 mg – placebo
**Predose**	0.8	1.8	−0.2	−0.6	1.0 [−0.1, 2.2]	2.1 [0.9, 3.3]	−0.3 [−1.5, 0.9]
**5 min**	6.0	11.9	3.7	1.5	2.2 [0.8, 3.6]	8.1 [6.8, 9.5]	−2.2 [−3.6, −0.8]
**10 min**	7.0	16.4	−0.6	−1.0	7.6 [6.3, 8.9]	17.0 [15.7, 18.3]	−0.4 [−1.7, 1.0]
**30 min**	5.0	12.5	−0.2	0.4	5.2 [3.8, 6.6]	12.7 [11.3, 14.0]	0.6 [−0.8, 2.0]
**1 h**	2.3	7.2	−0.4	2.0	2.7 [1.4, 4.0]	7.6 [6.3, 8.9]	2.4 [1.1, 3.7]
**2 h**	0.8	5.1	−1.1	0.2	1.9 [0.7, 3.0]	6.2 [5.0, 7.3]	1.3 [0.1, 2.4]
**4 h**	0.5	4.5	−0.9	−0.3	1.4 [0.3, 2.5]	5.4 [4.3, 6.6]	0.6 [−0.5, 1.7]
**8 h**	3.1	7.1	1.5	2.4	1.6 [0.4, 2.8]	5.7 [4.5, 6.8]	0.9 [−0.3, 2.1]
**12 h**	5.7	9.1	3.3	3.6	2.4 [1.2, 3.6]	5.7 [4.5, 6.9]	0.2 [−1.0, 1.4]

Abbreviation is as follows: CI, confidence interval limit.

#### Pharmacokinetics

Table 4 summarizes the PK data from the FF/VI study. Following administration of FF/VI via DPI (200/25 and 800/100 μg) for 7 days, the maximal plasma concentrations of FF were achieved, on average, at 64 and 123 min postdose, respectively. The maximal VI plasma concentrations were achieved, on average, at 5 and 6 min postdose, for FF/VI 200/25 and 800/100 μg, respectively. For both FF and VI, the intersubject coefficient of variation for all PK parameter estimates (AUC and *C*_max_) was generally high.

**Table 4:** Summary of selected FF and VI pharmacokinetic parameters following repeated (7 days) inhaled administration of FF/VI (200/25 and 800/100 μg; pharmacokinetics population)

	Parameter	Treatment (μg)	*N*	*n*	n*	Geometric mean (CV%)	95% CI
**FF**	**AUC_(0–24)_ (pg h ml^−1^)**	FF/VI 200/25	81	71	1	507 (37.9)	465, 553
		FF/VI 800/100	80	80	0	1921 (43.4)	1751, 2107
	**AUC_(0–_*_t_*_)_ (pg h ml^−1^)**	FF/VI 200/25	81	80	1	398 (90.1)	335, 472
		FF/VI 800/100	80	80	0	1927 (43.8)	1755, 2115
	***C*_max_ (pg ml^−1^)**	FF/VI 200/25	81	80	0	39.7 (35.7)	36.8, 42.9
		FF/VI 800/100	80	80	0	130 (32.3)	122, 140
	*T*_max_ (h)*	FF/VI 200/25	81	80	0	1.07 (0.08, 8.08)	NA
		FF/VI 800/100	80	80	0	2.05 (0.08, 8.08)	NA
**VI**	**AUC_(0–24)_ (pg h ml^−1^)**	FF/VI 200/25	81	57	2	85.0 (76.6)	71.0, 102
		FF/VI 800/100	80	74	0	775 (36.6)	714, 842
	**AUC_(0–_*_t_*_)_ (pg h ml^−1^)**	FF/VI 200/25	81	74	2	59.8 (77.7)	51.0, 70.2
		FF/VI 800/100	80	74	0	755 (40.1)	691, 826
	***C*_max_ (pg ml^−1^)**	FF/VI 200/25	81	74	1	115 (56.9)	102, 130
		FF/VI 800/100	80	74	0	527 (37.2)	485, 573
	*T*_max_ (h)*	FF/VI 200/25	81	73	1	0.083 (0.083, 0.550)	NA
		FF/VI 800/100	80	74	0	0.100 (0.083, 0.267)	NA

Abbreviations are as follows: AUC_(0–24)_, area under the concentration-time curve from time zero to 24 h post-dose; AUC_(0–t)_, area under the concentration-time curve from time zero to last time of quantifiable concentration; CI, confidence interval limit; *C*_max_, maximum observed concentration; CV, coefficient of variation; *N*, number of subjects treated; *n*, number of subjects with nonmissing observations (including imputable noncalculable values);

n* number of subjects for whom parameter cannot be derived because of noncalculable concentrations; NA, not applicable; and*T*_max_, time to maximum concentration.

* Median (range).

##### Fluticasone furoate/vilanterol PK-PD modelling

Scatter plots of FF exposure *vs*. QTcF (Supporting information, Figure S2A) and *vs*. heart rate (Supporting information, Figure S2B) showed no relationship between these parameters and FF plasma concentrations; therefore, no formal PK-PD analysis was conducted for FF.

In contrast to FF, the PD parameters of QTcF and heart rate displayed time course profiles that mirrored the median PK concentration profiles for VI, indicating that there may be a relationship between the PD effects seen and the plasma concentrations of VI. Significant transient effects had been seen for both QTcF and heart rate, so PK-PD modelling was conducted for VI *C*_max_. A clear baseline effect was observed for both QTcF and heart rate, and the final models included baseline as a significant covariate on the estimate of the intercept (Table 5). Over the range of values observed, both maximal QTcF and maximal heart rate were linearly related to VI *C*_max_. However, due to the limited effects observed, only shallow slopes (0.00751 ms pg^−1^ ml and 0.0173 beats min^−1^ pg^−1^ ml, respectively) were estimated from the modelling. Step-linear and log-linear models were also evaluated but showed no added value over the linear model.

**Table 5:** Summary of pharmacokinetic–pharmacodynamic modelling results for maximal QTcF and maximal heart rate in relationship to VI (pharmacokinetics population)

Parameter	Maximal QTcF		Maximal heart rate	
	Estimate [95% CI]	Precision (CV%)	Estimate [95% CI]	Precision (CV%)
**Intercept**	74.6 ms [40.1, 109.0]	23.6	23.6 beats min^−1^ [15.2, 32.0]	7.4
**Slope**	0.00751 ms pg^−1^ ml [0.0040, 0.0110]	25.0	0.01730 beats min^−1^ pg^−1^ ml [0.0150, 0.0200]	18.3
**Baseline covariate effect**	4.53 [1.98, 7.08]	28.7	1.86 [0.839, 2.880]	28.0
**Intersubject variability in intercept (%)**	0.446 [−0.177, 1.070]	71.3	0.0132 [−0.001, 0.270]	11.5
**Intersubject variability in baseline (%)**	NC	NC	0.485 [−0.354, 1.320]	88.2
**Residual error**	50.7 ms [39.2, 62.2]	712	24.2 beats min^−1^ [16.8, 31.6]	492

Abbreviations are as follows: CI, confidence interval limits; CV, coefficient of variation; and NC, not calculated.

Visual predictive checks for the *C*_max_/maximal QTcF model and the *C*_max_/maximal heart rate model (Supporting information, Figure S3) showed that the majority of the data were captured in the prediction interval that captured 90% of the population, as indicated by the 5th and 95th percentile boundary.

#### Safety and tolerability

Fluticasone furoate/vilanterol was well tolerated at both therapeutic and supratherapeutic strengths. There were no serious adverse events or subject withdrawals due to AEs. No AEs were severe and all resolved during the study. The majority of subjects (72%) reported AEs that were considered related to study medication, with the highest frequency seen in the FF/VI 800/100 μg group (51%) compared with placebo (24%) and similar incidences observed in the FF/VI 200/25 μg and moxifloxacin groups (40 and 41%, respectively). The most frequently reported AE related to study medication was headache, as follows: FF/VI 200/25 μg, 22%; placebo, 17%; FF/VI 800/100 μg, 16%; and moxifloxacin, 14%. The incidence of nausea was higher following treatment with moxifloxacin than the other treatment regimens (10 *vs*. 0–3%). The incidence of predefined AEs of special interest, i.e. those that were likely to be LABA related or ICS related, was greatest in the FF/VI 800/100 μg regimen, with palpitations (15 *vs*. 1–5%), oropharyngeal pain (8 *vs*. 0–1%) and tremor (8 *vs*. 0%) being the most frequently reported. Notably, only one subject (1%) reported palpitations in the FF/VI 200/25 μg group. No clinically significant ECG abnormalities were recorded during the study.

### Fluticasone furoate TQT study data

#### Demographics

Table 1 summarizes the subject disposition and demographics for the FF TQT study, which were similar to those enrolled in the FF/VI study. One subject withdrew due to personal reasons having received placebo, moxifloxacin and FF.

#### Pharmacodynamics

##### Effect of FF and moxifloxacin on QTcF

**Figure 3:**
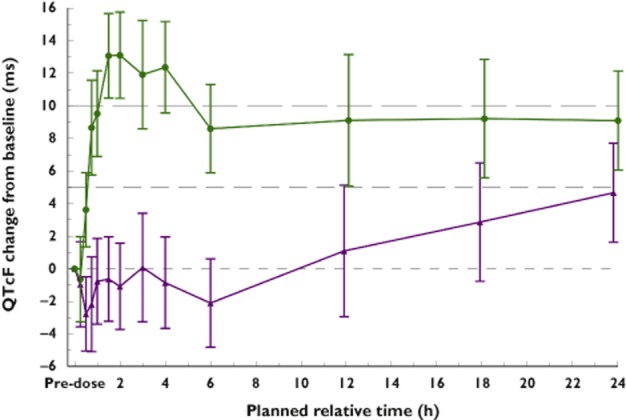
QTcF (Fridericia's correction) adjusted mean (90% CI) change from baseline (difference from placebo) 0–24 h after single dose FF (4000 μg) and single dose moxifloxacin (400 mg) ('all subjects' population). 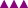, FF 4000 μg; 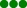, Moxi 400 mg

Lack of effect on QTcF was observed following a single supratherapeutic dose of FF (4000 μg); all upper 90% CI limits were <10 ms for mean change from baseline at each time point (Figure 3).

Single-dose moxifloxacin (400 mg) produced similar observations to those seen in the FF/VI study, with QTcF prolongation observed at all time points from 45 min postdose, and the highest mean change from baseline was 13.1 ms at 2 h postdose (Figure 3).

##### Categorical QTcF analysis

Categorical analysis of QTcF did not produce absolute QTcF values >450 ms or changes in QTcF values >30 ms.

The results for single-dose moxifloxacin were similar to those seen in the FF/VI study, with absolute QTcF values >450 ms in a minority of subjects (3%); no values were >480 ms. Changes in QTcF over 30 ms relative to baseline were observed in between one and six subjects (3–15%) at various time points over 24 h.

##### Heart rate

In comparison with placebo, increases in mean maximal heart rate (0–24 h) of 1.6 and 0.5 beats min^−1^ were observed for fluticasone furoate 4000 μg and moxifloxacin 400 mg, respectively.

#### Pharmacokinetics

After single doses of supratherapeutic FF (4000 μg) via Diskus™/Accuhaler™, the maximal concentration (358.2 pg ml^−1^) was achieved at 30 min postdose, with an AUC_(0–∞)_ of 6407.3 pg h ml^−1^.

#### Safety and tolerability

Single doses of FF 4000 μg were also well tolerated, with no serious adverse events or significant AEs observed.

## Discussion

The results of this study demonstrate that repeat-dose FF/VI 200/25 μg, administered once daily for 7 days, was not associated with QTc changes of concern as defined by the ICH E14 guidelines; increases in time-matched mean QTcF or QTci were <5 ms compared with placebo, with all 90% CIs being <10 ms [18,19]. At a fourfold higher FF/VI strength (800/100 μg) there was a small, transient effect on QTcF during the first hour after dosing (with the upper 90% CI >10 ms at one time point), but no significant effect on QTci was observed; all mean time-matched differences from placebo were <5 ms (90% CIs <10 ms). This indicates that the ‘positive’ effect on the QT interval observed with FF/VI 800/100 μg, according to the ICH guidelines, was dependent on the QTc correction factor applied rather than indicating a clinically significant effect of FF/VI on the QTc interval, *per se* [18,19].

The uncorrected QT interval shortens with increasing heart rate [32]. The corrected QT (QTc) interval represents the QT value that would be noted in the same ECG at a heart rate of 60 beats min^−1^ [25]. Many QT correction methods are available, and selection of the most appropriate QT interval correction factor is a topic of considerable controversy and debate [25,^33^,34]. Correction using either Bazett's formula (QTcB =QT/(R–R)^1/2^) or Fridericia's formula (QTcF = QT/(R–R)^1/3^) is well established but assumes a fixed exponential QT:RR relationship that is applicable to all subjects [23,^24^,35]. However, QT/RR relationships have substantial intersubject variability with high intrasubject stability, and the hypothesis that a single mathematical formula can describe a ‘physiological’ relationship that is applicable to all subjects is incorrect [25,26]. Ideally, individualized correction formulae (QTci) based on drug-free data collected over a range of heart rates from each subject should be used to assess drug-related QT prolongation [33]. For confidence in the estimation of QTci, the number of drug-free ECGs obtained and used should be as large as possible; at least 20–50 per subject [35].

Drugs with inherent heart rate effects are much more challenging to study in TQT studies; there is limited experience with such compounds and there is no firm guidance on which correction factor to select [34]. When feasible, crossover designs including negative controls (i.e. placebo) are preferable for TQT studies because they allow determination of individual QT/RR relationships for each subject and the calculation of QTci [33]. In the present study, increases in heart rate were anticipated at the supratherapeutic FF/VI strength of 800/100 μg and consequently, individually corrected QT (QTci) interval data were also collected. The number of drug-free ECGs obtained (72) exceeded the minimum recommended [35]. At 800/100 μg, FF/VI did increase mean maximal heart rate, with the greatest difference compared with FF/VI 200/25 μg of approximately 9 beats min^−1^ seen 10 min postdose. Despite this difference in heart rate, time-matched QTci values were comparable between FF/VI 800/100 and 200/25 μg, suggesting that QTci represented a more suitable QT correction factor than QTcF for the data in this study. Furthermore, this would appear to be supported by the moxifloxacin data, where there was no effect on heart rate and the effects on QTcF and QTci were comparable. These data suggest that the increases in QTcF seen at the FF/VI 800/100 μg strength were a consequence of inadequate QT correction at the heart rates seen rather than an effect on the QT interval, *per se*. The lack of effect of FF/VI 800/100 μg on QTci according to the ICH E14 guidelines, despite the increases in heart rate, indicates that FF/VI was not associated with QTc prolongation at the dose administered [18,19]. A lack of effect of FF/VI on the QTc interval was also supported by the categorical analysis of postdose changes in QTcF, where increases of ≥30–60 ms were observed in only three individuals (4%) after FF/VI 800/100 μg.

The increases in heart rate recorded in this study following administration of FF/VI 800/100 μg were greater than those previously reported with VI 100 μg in healthy subjects, subjects with asthma or subjects with COPD [36]. Furthermore, more subjects reported AEs of palpitations following administration of FF/VI 800/100 μg (15%) compared with FF/VI 200/25 μg (1%). Given that a single supratherapeutic FF dose of 4000 μg did not affect heart rate, these effects of FF/VI were attributed to VI. Thorough QT studies are typically conducted in healthy subjects unless the drug under evaluation is unsuitable for administration in this population [18,19]. β_2_-Agonists have a class-related potential for cardiovascular effects [16,17], while cardiovascular disease is a known co-morbidity in subjects with COPD and is a leading cause of mortality in this population [37]. Consequently, the potential cardiovascular effects of VI are of particular clinical interest. Vilanterol at doses up to 100 μg has been generally well tolerated in healthy subjects (repeat dosing) and in subjects with COPD or asthma (single doses), with a low overall incidence of AEs compared with placebo, and few AEs potentially indicative of LABA-class effects (such as tremor, muscle twitching and palpitations). In addition, these VI doses were not associated with clinically significant abnormalities in vital signs, 12-lead ECG, Holter ECG, blood glucose or potassium [36]. No QTc effects were seen at VI doses up to 50 μg and, while some QTc effects were seen at 100 μg in healthy subjects and in subjects with asthma, no significant QTc effects were seen in subjects with COPD [36]. These data were in agreement with those of the 28 day dose-ranging (phase II) VI studies (VI 3–50 μg) in subjects with asthma [8] and in subjects with COPD [10], in which no significant QTc effects were seen, and only minor effects on heart rate were recorded (mean increases of 2 beats min^−1^ or less). In these studies, the incidence of AEs potentially indicative of an LABA-class effect were <3% in any treatment group, indicating that VI is well tolerated in subjects with COPD or asthma at doses up to 50 μg [8,10]. Likewise, in large-scale (phase III) 6 month studies in subjects with COPD [38,39] or in 12 month studies in subjects with asthma [40], VI 25 μg administered as FF/VI or VI alone (COPD only) was also not associated with clinically significant cardiovascular effects, including effects on the QTc interval. The reasons for the more marked effects on heart rate seen in healthy subjects in the present study are not known. Systemic VI exposure (*C*_max_) is greater in healthy subjects than in subjects with either asthma or COPD [36], which may account for the lack of effect of VI on heart rate in these patient groups. Consequently, the heart rate effects seen in this study in healthy subjects, particularly at the FF/VI 800/100 μg dose, are not considered to be representative of effects observed with VI 25 μg in clinical use.

No formal PK-PD modelling was performed with FF owing to the apparent absence of a relationship between FF and QTcF or heart rate following graphical evaluation of FF exposure (*C*_max_) and these parameters. The later *T*_max_ observed at the higher dose of FF (800 μg) was attributed to the known absorption-rate-limited pharmacokinetics of inhaled FF [41]. The differences in *T*_max_ between FF (800 μg) in the main study and FF (4000 μg) in the supportive FF TQT study were not unexpected given the different formulations and inhalers used in the two studies. The PK-PD modelling demonstrated that VI exposure (*C*_max_) was related to increases in both heart rate and QTcF. However, the slopes for the relationships were extremely shallow. Based on the VI *C*_max_ value for FF/VI 200/25 μg for healthy subjects in this study (115 pg ml^−1^), these would represent an increase in mean maximum heart rate of approximately 2 beats min^−1^ and an increase in mean maximal QTcF of approximately <1 ms. Lesser effects would be anticipated in subjects with asthma or COPD based on the lower VI systemic exposure previously described.

A potential limitation of this study is that it did not include a direct comparison with another established long-acting β-adrenoceptor agonist, such as salmeterol or formoterol. These compounds were developed prior to establishment of the requirements for a TQT study [18,19] and so comparable data to the present study are not available. Although class-related QT prolongation is described for β_2_-agonists, the studies reported typically used Bazett's correction, which overestimates QTc when heart rate increases, and consequently, a systematic bias may have implicated these drugs in cardiac prolongation [17]. More recently, a lack of effect on QTcF has been reported with the LABA indacaterol using a parallel-group study design [42], although the highest dose examined (600 μg) represented only a twofold multiple of the highest approved dose in the EU [43].

Another potential limitation of this study is that FF and VI were only administered in combination, and the QTc effects of the separate components were not studied. However, in the supporting study, high single-dose FF (4000 μg) did not produce QTc prolongation, which is in agreement with the fact that corticosteroids are not associated with this effect [28]. There was no relationship observed between the QTcF interval and FF exposure over a wide range of exposures, including those considerably above the therapeutic range. Co-administration of FF with VI does not significantly affect VI pharmacokinetics [44]. Consequently, the effects of FF/VI on the QTc interval are considered to be attributable to the VI component and indicative of the QTc effects of VI administered alone at the same doses.

Single-dose moxifloxacin (400 mg) was included as a positive control in the present study in accordance with guidelines [18,19]. The magnitude, time course and duration of the increase QTcF was comparable to that reported in a meta-analysis of several TQT studies [45] and clearly demonstrated assay sensitivity according to the established criteria [34].

Thorough QT studies should ensure that dose–response and concentration–response relationships for QTc prolongation are fully characterized, including exploration of concentrations higher than those anticipated in therapeutic use [18,19]. Fluticasone furoate and VI are both extensively metabolized [46,47], and high systemic exposure could occur at therapeutic doses in conditions of metabolic impairment. In the present study, a high FF/VI strength (800/100 μg) was selected, which represented a fourfold multiple of the proposed highest therapeutic strength (200/25 μg) and produced systemic VI exposure that is likely to be considerably above that seen in subjects with asthma or COPD, particularly as VI exposure (*C*_max_) is lower in these patient groups than in healthy subjects [36]. Systemic exposure to FF and VI following administration of FF/VI 800/100 μg was also greater than would be anticipated following administration of the highest therapeutic strength of FF/VI (200/25 μg) in conditions of metabolic impairment in subjects with mild, moderate or severe hepatic impairment, severe renal impairment, or in healthy subjects administered the strong CYP3A4 inhibitor ketoconazole [48,49]. These data provide reassurance that a lack of effect of FF/VI on the QTc interval would be anticipated in clinical use.

In conclusion, the results of this study indicate that FF/VI at a therapeutic strength of 200/25 μg does not have a significant effect on the QTc interval as measured by either QTcF or QTci. Fluticasone furoate/vilanterol, at a fourfold multiple of this strength (800/100 μg), was associated with transient heart rate changes and produced a small, predictable and transient effect on QTcF during the first hour after dosing. However, there was no significant effect of FF/VI 800/100 μg on QTci, indicating that the effects observed at this strength were dependent on the QT correction method applied rather than the effect on the QTc interval, *per se*.

## Competing Interests

These studies were funded by GlaxoSmithKline: study number FFR101888 (trial conducted prior to http://ClinicalTrial.gov registration requirements) and study number HZA102936 (http://ClinicalTrials.gov identifier NCT01209026). All authors have completed the Unified Competing Interest form and declare that RK, AA, KK and CC are employees of and hold stock in GlaxoSmithKline with no other relationships or activities that could appear to have influenced the submitted work. PS was a paid contractor of GlaxoSmithKline for this study.

The authors would like to thank: The staff at the Hammersmith Medicine Research (HMR) Unit (Park Royal Hospital, London, UK), in particular Kate Hanrott (Project Manager) and investigators, Dr Steve Warrington and Dr Benjamin van Hecke for their help in the conduct of the FF/VI TQT study and the staff at Parexel, Northwick Park Hospital, Harrow, UK for their help in the conduct of the FF TQT study; staff in the Department of Worldwide Bioanalysis, GSK, Ware and at ICON Development Solutions (Ellicott City, MD, USA) for PK analysis support; and Dr Kathryn White (of Cathean Limited Medical Writing Consultancy) for her assistance in the writing of this paper.

## Supporting Information

**Figure S1** Scatter plots for QT vs. RR interval. Scatter plots for uncorrected QT (A), QTcB (B), QTcF (C) and QTci (D) for FF/VI **Figure S2** Scatter plots for time-matched difference from placebo in change from baseline QTcF vs. FF concentration (800, 200 and 4000 μg) 0–24 h after dosing on day 7 (A) and timematched difference from placebo in change from baseline heart rate *vs.* FF concentration (800, 200 and 4000 μg) 0–24 h after dosing on day 7 (B; per protocol population) **Figure S3** Visual predictive checks (VPCs) for the maximal QTcF *vs. C_max_* model (A) and the maximal heart rate *vs. C_max_* model(B).In both panels, the red lines represent the 5th and 95th percentiles of the model simulation; the blue line represents the predicted median (PK population)

Table S1 Studies used to calculate the sample size for the FF/VI TQT study using an integrated analysis

Appendix S1 Study methods (subject compliance) and statistical analysis (sample size calculation: FF/VI TQT study)
