# Supplementary figures and images for: A repeat-dose thorough QT study of inhaled fluticasone furoate/vilanterol combination in healthy subjects
Source: Br J Clin Pharmacol. 2014 Feb 21;77(3):466–79. doi: 10.1111/bcp.12243 (PMC3952721; doi:10.1111/bcp.12243)

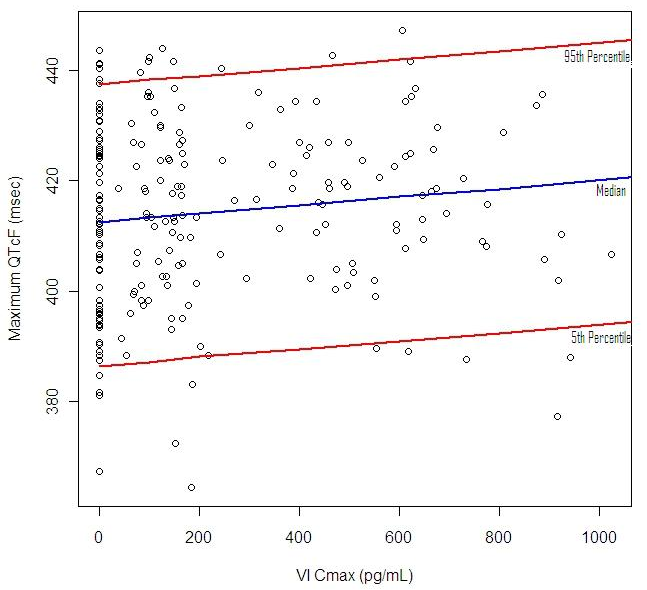

Supplement: Supplementary file 1 — Figure S1 Scatter plots for QT vs. RR interval. Scatter plots for uncorrected QT (A), QTcB (B), QTcF (C) and QTci (D) for FF/VI Figure S2 Scatter plots for time-matched difference from placebo in change from baseline QTcF vs. FF concentration (800, 200 and 4000 μg) 0–24 h after dosing on day 7 (A) and timematched difference from placebo in change from baseline heart rate vs. FF concentration (800, 200 and 4000 μg) 0–24 h after dosing on day 7 (B; per protocol population) Figure S3 Visual predictive checks (VPCs) for the maximal QTcF vs. Cmax model (A) and the maximal heart rate vs. Cmax model(B).In both panels, the red lines represent the 5th and 95th percentiles of the model simulation; the blue line represents the predicted median (PK population) [file bcp0077-0466-sd1.zip › bcp_12243_sup_0007_figureS3a.TIF]

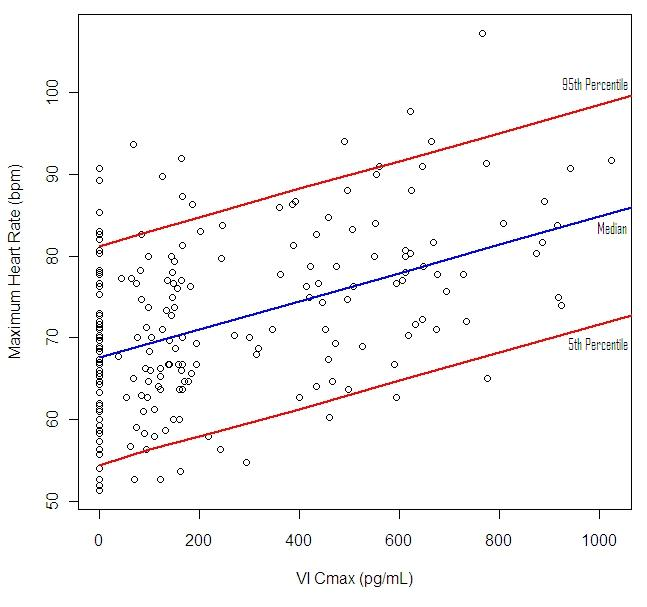

Supplement: Supplementary file 1 — Figure S1 Scatter plots for QT vs. RR interval. Scatter plots for uncorrected QT (A), QTcB (B), QTcF (C) and QTci (D) for FF/VI Figure S2 Scatter plots for time-matched difference from placebo in change from baseline QTcF vs. FF concentration (800, 200 and 4000 μg) 0–24 h after dosing on day 7 (A) and timematched difference from placebo in change from baseline heart rate vs. FF concentration (800, 200 and 4000 μg) 0–24 h after dosing on day 7 (B; per protocol population) Figure S3 Visual predictive checks (VPCs) for the maximal QTcF vs. Cmax model (A) and the maximal heart rate vs. Cmax model(B).In both panels, the red lines represent the 5th and 95th percentiles of the model simulation; the blue line represents the predicted median (PK population) [file bcp0077-0466-sd1.zip › bcp_12243_sup_0008_figureS3b.tif]
