# Supplementary material for: A repeat-dose thorough QT study of inhaled fluticasone furoate/vilanterol combination in healthy subjects
Source: Br J Clin Pharmacol. 2014 Feb 21;77(3):466–79. doi: 10.1111/bcp.12243 (PMC3952721; doi:10.1111/bcp.12243)
Supplement: Appendix S1 — Study methods (subject compliance) and statistical analysis (sample size calculation: FF/VI TQT study) [file bcp0077-0466-sd3.pdf]

British Journal of Clinical Pharmacology

Supplemental Information

Methods

FF/VI TQT study

Subject Compliance

Subject compliance with dosing was achieved through a combination of supervised dosing and the use of a dose counter on the inhaler. All subjects attended the study unit on Day -1 of all treatment periods and stayed in the unit overnight. On Day 1 of each treatment period dosing was supervised and subjects remained in the unit until approximately 1 h post-dose. All subjects self-administered the doses on Days 2-6 and the time and date of each dose were recorded in a patient diary. When the subject returned to the study unit the pharmacist or study nurse recorded the number displayed on the inhaler dose counter. This was checked by the study monitor and recorded to verify that the medication had been taken as per protocol. Subjects returned to the unit on the evening of Day 6, the Day 7 dose was supervised and the subjects remained in the unit until all the 0-24 hour post-dose assessments were completed. In total 85 subjects were enrolled and randomised and 77 (91%) completed the study as planned (i.e. they completed all four treatment arms). Eight subjects were withdrawn from the study prematurely and were not included in the PP Population. A further 4 subjects completed all four treatment arms but each one missed a Day 7 dose in one treatment period only and were also not included in the PP Population. Consequently a total of 12 subjects were excluded from the PP population. The compliance checks, performed as described, enabled these discrepancies to be identified and documented.

Statistical Analysis

The sample size calculation for the FF/VI TQT study was performed using an integrated analysis from multiple studies with VI. These studies are currently unpublished. However, information about these studies can be found at www.clinicaltrials.gov using the NCT numbers provided in Supplemental Table 1. Study B2C10001 was not registered on

www.clinicaltrials.gov, but information relating to this study and the other studies listed, can be found on the GlaxoSmithKline Clinical Trials Register at www.gsk-clinicalstudyregister.com.

FF TQT study

Subject Compliance

All study medication (single dose study) was administered by an authorised member of the site personnel and compliance was verified using the drug accountability records.

Tables

Supplemental Table 1: Studies Used to Calculate the Sample Size for the FF/VI TQT Study Using an Integrated Analysis

Study Number	NCT Number
B2C10001	NCT – not available
B2C101762	NCT00354874
B2C106093	NCT00347139
B2C108562	NCT00372112
B2C104604	NCT00381667
B2C108784	NCT00469040
B2C106996	NCT00463697
B2C110165	NCT00519376
HZA105871	NCT00538057
HZA102940	NCT00625196

British Journal of Clinical Pharmacology
